# Research on proactive defense and dynamic repair of complex networks considering cascading effects

**DOI:** 10.1038/s41598-024-61188-y

**Published:** 2024-05-08

**Authors:** Zhuoying Shi, Ying Wang, Haijuan Li, Gang Feng, Chaoqi Fu

**Affiliations:** 1 School of Air Defense and Missile Defence, Air Force Engineering University, Xi’an, 710051 People’s Republic of China; 2 School of ATC Navigation, Air Force Engineering University, Xi’an, 710051 People’s Republic of China; 3 School of Equipment Management and UAV Engineering, Air Force Engineering University, Xi’an, 710051 People’s Republic of China

**Keywords:** Cascade effect, Proactive defense, Repair strategy, Marginal nodes, Statistics, Complex networks

## Abstract

Cascading effects can result in the nonlinear propagation of failures in complex networks, ultimately leading to network collapse. Research on the fault propagation principles, defense strategies, and repair strategies can help mitigate the effects of cascading failures. Especially, proactive defense and dynamic repair are flexible and effective methods to ensure network security. Most studies on the cascade of complex networks are based on the unprocessed initial information of the network. However, marginal nodes are a type of node that cloaks the initial information of the network. In this study, we rank the importance of nodes according to the intensity of network energy confusion after the removal of this node, clarify the meaning of marginal nodes and proposed two methods to screen marginal nodes. The results indicated that the proactive removal of marginal nodes can effectively reduce the effect of cascading failures without causing any negative disturbance to the energy flow of the network. In addition, network repair according to the proposed strategy can minimize the cascade effect in the repair process and improve repair efficiency.

## Introduction

Complex network theory provides new perspectives and methods for studying the complexity of the world. Complex systems can often be characterized by networks, which is beneficial for studying the relationships and interactions between systems, and have achieved fruitful results in many areas^1–4^. Strogatz et al.^2^ and Barabasi et al.^3^ first discovered the laws of complex system structures from topological properties, such as small-world, scale-free, and self-similar properties. Subsequently, a large number of scholars from different fields confirmed the universality of these laws^2,3,5^. Topology is just one aspect of the complexity of complex systems^6,7^. When considering the role of energy flow in a network, cascading effects^8,9^ are a crucial aspect that help us understand how small errors in complex systems can cause major damage. Cascading faults have caused huge disasters in transportation networks, power grids, information networks and other fields^10–12^. For example, the blackout under the influence of the snow disaster in southern of China in 2008 had a major impact on people's lives and caused huge economic losses. Therefore, cascading effects have attracted considerable attention from scholars.

Previous research on the cascade effect can be divided into two categories: one involves developing the theoretical model and studying the working principles^13–15^; the other focuses on reducing the impact of fault diffusion by optimizing the network layout and designing effective strategies^16,17^. Understanding the ability of each node to influence the energy flow in the network is critical, which can help to realize the optimal design of the network and formulate effective coping strategies. We determined that a class of nodes—marginal nodes, the removal of such nodes will not change the flow path of energy flow in the network. The marginal node has a weak influence on the disturbance of energy in the network, so they are often overlooked and has not been accurately understood. In the research results of network robustness^18,19^, nodes that meet the definition of marginal nodes are usually ranked lower in importance under the network robustness index and are often ignored. However, some studies have indicated that marginal nodes have an impact on blocking the spread of cascading faults in the network. Adilson et al.^20^ discovered that the impact of a cascading failure could be effectively reduced by proactively removing the right amount of nodes and edges before the cascading impact occurs. Fu et al.^21,22^ studied the dynamic repair and proposed the effective strategies to avoid the secondary failure of the repaired nodes affected by cascading, significantly improving repair efficiency. Proactively removed nodes conform to the definition of marginal nodes in this paper, and dynamic repair rules are also associated with marginal nodes, which will be explained in detail in the following subsections. Therefore, although the marginal node is not clearly recognized initially, it does significantly reduce the impact of cascading effects^20–23^. However, the mechanism of action of marginal nodes in the process of network defense and repair remains unclear. Changes in the network structure resulting from network damage or repair cause network energy flow fluctuations, which is the primary cause of cascade failure. Regardless of the implementation of proactive defense or dynamic repair, the order of nodes being removed or repaired will affect the strength of network energy flow fluctuations. Effective control of energy flow fluctuations is the key to strategy implementation. However, all these strategies are based only on unprocessed information in the original network, marginal nodes make information on the original network unreliable and require further processing. The proactive removal of marginal nodes can effectively reduce the effect of cascading failures without causing negative disturbance to the energy of the network. Therefore, screening marginal nodes and correctly understanding the ability of nodes to influence energy flow can effectively improve the defense capability and repair efficiency of the network.

The remainder of this paper is organized as follows: In "Globally distributed cascading failure model" section, we introduce the cascading failure model and analyze the effect of node removal on the network energy flow; in "Marginal node screening and importance ranking" section, we propose two methods to screen the marginal nodes and sort all nodes; in "Simulation analysis and application"Section, we simulate the effects of the proposed proactive defense and dynamic repair strategies under the influence of cascades and analyze their working principles; and finally, conclusions are presented in "Conclusion"Section.

## Globally distributed cascading failure model

Assuming $G = (V,\;E)$ is an undirected graph, $V = \{ v_{1} ,v_{2} , \cdots ,v_{n} \}$ is a set of nodes, and $E = \{ e_{1} ,e_{2} , \cdots ,e_{m} \}$ is a set of edges. Here we introduce the globally distributed cascading failure model based on load dynamics. Each time step one unit of energy flow (such as the current in the grid, the traffic flow in the transportation network) is sent from node $i$ to node $j$, and the shortest path is preferred. Let $L_{k}^{ij}$ denote the contribution of the ordered pair $(i,j)$ to the load of node $k$. If the shortest path of ordered pair $(i,j)$ goes through node $k$, then $L_{k}^{ij} = 1$, otherwise $L_{k}^{ij} = 0$. If multiple shortest paths are available, the energy flow is divided evenly. Therefore, the load of node $k$ is defined as $L_{k} = \sum\limits_{i,j} {L_{k}^{ij} }$, which reflects the total energy flow passing through this node per unit of time. The capacity of a node is the maximum load that the node can handle, which has a linear relationship of $C_{k} = (1 + \alpha )L_{k} { = }L_{k} { + }\Delta C_{k}$ with the initial load, where $\alpha > 0$ is the tunable capacity parameter, $\Delta C_{k} = \alpha L_{k}$ is the redundancy capacity, which indicates the ability to resist the network energy shock. Each node in a normal network satisfies $L_{k} < C_{k}$ in a free-flowing state of energy flow. If a node is attacked and failed, the shortest path between some pairs of nodes may be changed, resulting in a global redistribution of load among the remaining nodes in the network. This type of energy confusion easily leads to cascade failure. If $L_{k} > C_{k}$, then node $k$ fails due to overload, which is reflected in changes in network structure caused by removal from the network. This will lead to the change of the energy flow path between the remaining pairs of nodes in the network, resulting in the redistribution of the network load and the cascading fault propagation.

The load generated by node $k$ in the network is^20^:

1 $$L_{k}^{g} = \sum\limits_{j = 1,j \ne k}^{N} {(D_{kj} - 1)} = (\overline{D}_{k} - 1)(N - 1).$$

where $\overline{D}_{k}$ is the average shortest path length from node $k$ to all nodes of the network. Here, $L_{k}^{g}$ is proportional to the number of nodes *N* in the network. The total load of the network satisfies $TL_{N} = \sum\nolimits_{k} {L_{k} } = \sum\nolimits_{k} {L_{k}^{g} }$. When node $k$ is removed and no other nodes leave the network, the change in the total load of the network is expressed as follows:

2 $$\begin{aligned} \Delta TL & = TL_{N} - TL_{N - 1} = \sum\limits_{i = 1}^{N} {\left( {\sum\limits_{j = 1,j \ne i}^{N} {(D_{ij} - 1)} } \right)} - \sum\limits_{i = 1}^{N - 1} {\left( {\sum\limits_{j = 1,j \ne i}^{N} {(D_{ij}{\prime} - 1)} } \right)} \\ & = \sum\limits_{i = 1}^{N - 1} {\left( {\sum\limits_{j = 1,j \ne i}^{N - 1} {(D_{ij} - D_{ij}{\prime} )} } \right)} + 2L_{k}^{g} . \\ \end{aligned}$$

where $D_{ij}$ and $D_{ij}{\prime}$ are the shortest path length of node $i$ and node $j$ before and after node $k$ is removed. Here, $\sum\limits_{i = 1}^{N - 1} {\left( {\sum\limits_{j = 1,j \ne i}^{N - 1} {(D_{ij} - D_{ij}{\prime} )} } \right)}$ expresses the change of production load of the remaining nodes before and after node $k$ is removed, it is mainly reflected in the change of the shortest path length between the remaining nodes of the network. This part of the change is the source of the cascade failure. $2L_{k}^{g}$ represents the decrease in the total load of the remaining nodes in the network due to the removal of node k. This part of the load is not be redistributed, so that the change can be seen as a total increase in the redundant capacity of the remaining nodes in the network from another perspective. A network of $N$ nodes has a total of $2C_{N}^{2}$ energy flows. The load of node $i$ is $L_{i}$, which indicates number of $L_{i}$ energy flows through node $i$. Therefore, when a random node $k$ is removed, it can be equivalently considered that the increased redundant capacity of node $i$ is $L_{k}^{g} L_{i} /C_{N}^{2}$. Thus, the larger the initial load is, the greater the increased redundant capacity is.

## Marginal node screening and importance ranking

**Figure 1:**
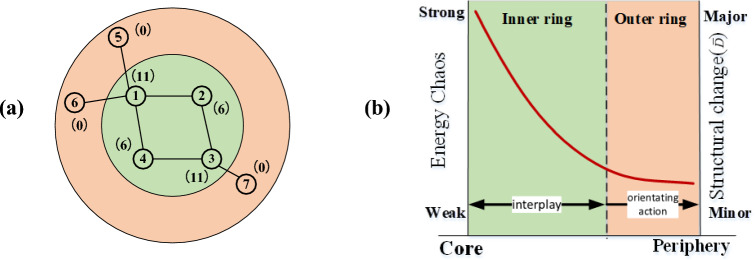
(**a**) Schematic of network information disguised by marginal nodes (the numbers in parentheses indicate the load of the node); (**b**) Effect of sorted nodes on energy confusion and structural changes.

Many studies were based on the initial information of the network. However, under the influence of marginal nodes, a layer of camouflage is inserted in the initial information of the network. For example, if node $v_{2}$ is attacked and removed in Fig. 1a, the load of node $v_{4}$ is doubled because of the redistribution load, node $v_{4}$ will fail due to cascading effects if $\alpha < 1$. However, if node $v_{1}$ is failed and removed, nodes $v_{5}$ and $v_{6}$ are off the network, failure because of overload does not occur. The load of node $v_{1}$ is considerably larger than that of node $v_{2}$, but after removal, the redistribution load generated by node $v_{2}$ is considerably larger than node $v_{1}$. The root cause is that after node $v_{1}$ is removed, nodes $v_{5}$ and $v_{6}$ are disconnected from the network. This effect can be considered that the separation of nodes $v_{5}$ and $v_{6}$ from the network lead to the redundant capacity of the remaining nodes in the network is indirectly increased, and the amount of redistributed load caused by node $v_{1}$ is not the original load $L_{1}$. The energy flow through node $v_{1}$ between nodes $v_{5}$, $v_{6}$ and other nodes in the network needs to be removed. This is why node $v_{1}$ has less impact than node $v_{2}$. Here nodes $v_{5}$ and $v_{6}$ are marginal nodes; they camouflage the load of node $v_{1}$ that is actually participating in the redistribution.

In general, if node $v_{k}$ is removed and causes number of $r$ nodes to leave the network. This process can be divided into two steps: (1) first remove these $r$ that are off the network, so that $DL = 2r\sum\limits_{j = 1}^{N - r} {D_{kj} }$ load is eliminated from the remaining nodes in the network (contains node $v_{k}$); (2) remove node $v_{k}$, and thus, the change in the total load of the network at this time is expressed in Eq. (2) and the redistribution load caused by the removal of node $v_{k}$ is $RL = L_{k} - 2r(N - r - 1)$, which is much lower than the results $L_{k}$ obtained under the initial network information.

**Figure 2:**
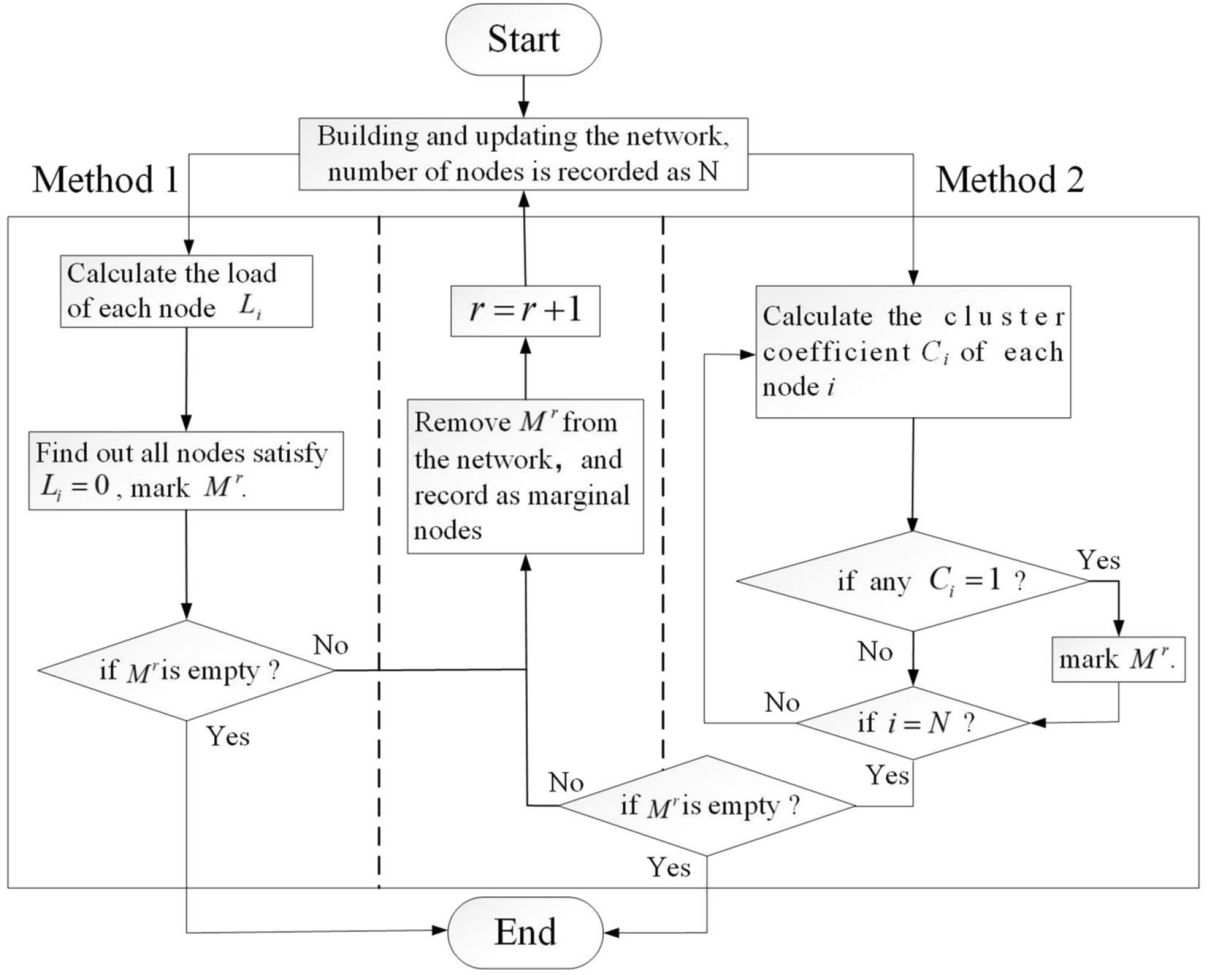
Flowcharts for screening marginal nodes.

The camouflage of the network by the marginal nodes leads to an inaccurate understanding of the real information of each node, which has a negative impact on the effectiveness of decision-making. Therefore, in order to obtain the real information of each node in the network, preprocessing is required. Two alternative methods for screening marginal nodes are depicted in Fig. 2. Method 1 is similar to the K-shell method. First, the nodes that are not on the shortest path of any node pairs ($L_{i} = 0$) are screened, and then these nodes are removed from the network. As these nodes are removed, the load ($L_{i}^{g}$) they generate in the network also disappears and does not affect the path of energy flow between other node pairs, which means that there is no energy confusion appears. These nodes have the same effect with respect to causing network energy confusion; therefore, they are all marked at the same level and arranged in descending order of node degree. The second step is to repeat the aforementioned operation until all nodes in the network are under load. The importance of the screened nodes increases layer by layer, resulting in layering.

**Figure 3:**
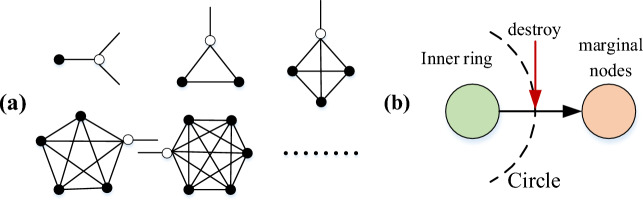
(**a**) Schematic of the fully coupled structure at each stage (Solid circle nodes are marginal nodes); (**b**) Mechanism of inner ring nodes degenerating into marginal nodes.

After processing, the nodes in the network are divided into two categories. Marginal nodes are the removed nodes, and their characteristic is that they will not cause energy confusion when they are removed from network in the current stage. The hierarchical relationship formed by the marginal nodes is recorded as the outer ring. The remaining nodes form the inner ring. One of the characteristics of these marginal nodes is that their topology is fully coupled; that is, the node and its neighboring nodes are connected to each other, which satisfy that the cluster coefficient $C_{i} = 1$^1,24^, as depicted in Fig. 3a. Therefore, Method 2 depicted in Fig. 2 can be used to screen the fully coupled nodes in the network for determining the marginal nodes, which has more advantages in large-scale networks compared to Method 1. Analysis indicates that the nodes in the inner ring must be on the circuit. If the circuit is broken, some nodes are automatically converted to marginal nodes. The relationship between marginal nodes and the nodes of the inner ring is illustrated in Fig. 3b. After removing all the marginal nodes, the load of remaining nodes recorded as New-load, which can be used to determine the intensity of energy confusion caused by the removal of the node. These nodes are sorted in the descending order in the New-load. The combination of the inner ring and outer rings is displayed in Fig. 1b. Thus, the nodes were sorted according to the relationship of network energy confusion caused by node removal, and the higher the impact, the higher the ranking. The outer ring is a one-way relationship, in which the load redistribution caused by the low-ranked nodes does not affect the higher-ranked nodes. However, the effect of the redistribution load caused by nodes of inner rings is mutual; that is, the higher the node's ranking is, the stronger the energy confusion caused to the network is when the node is removed. It should be noted that energy confusion is not exactly equivalent to cascading failure.

The cloaking of the initial information of the network introduced by the marginal nodes can confuse the enemy and improve the survivability of the network. The proactive removal of the marginal nodes can effectively mitigate the cascade effect without any risk. In the dynamic repair process, appropriate handling of marginal nodes can also effectively improve network repair efficiency.

## Simulation analysis and application

### Proactive defense

We considered a random scale-free networks (SFNs) with a total number of nodes $N = 1000$ and average connectivity degree of $\left\langle k \right\rangle \approx 4$, and focused on global cascades triggered by initial attacks on a small fraction $\rho = 0.001$ of most loaded nodes. The simulation is based on SFNs and it is hoped that similar results will also be applicable to other networks with heterogeneous distribution of loads. Each value was obtained by averaging results from 20 independent networks.

It is an an effective method of active defense that when the network is attacked, some nodes are proactively removed to reduce the impact of cascading failures and achieve the purpose of reducing network damage. The proactive removal strategy in this paper is removing the marginal nodes layer by layer from the outer layer to the inner layer.

**Figure 4:**
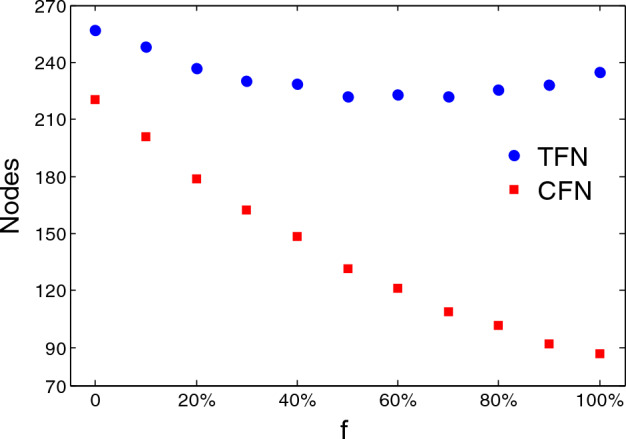
Failed nodes as a function of the fraction $f$ of nodes proactively removed.

Nodes are removed from the network because of two reasons: one reason is failure caused by attacked or energy overload; another reason is that the node loses its connection to the network (such as nodes $v_{5}$ and $v_{6}$). Therefore, we recorded two results: the number of cascading failed nodes (CFNs) and the total number of failed nodes (TFNs). Here, $f$ is the ratio of the number of proactively removed marginal nodes to the all of marginal nodes. In the initial stage, with the increase of proactive removal of marginal nodes, the cascading effect was alleviated and the TFNs decreased, as depicted by the solid circles in Fig. 4. When the number of proactively removed nodes exceeds the critical point, the damage caused by proactive removal exceeds the cascading effect, the TFNs increase again. However, the number of nodes that failed because of overload decreased with the number of proactively removed marginal nodes increased, as depicted in the solid square. This phenomenon indicates that the proactive removal of marginal nodes reduced the effect of redistribution of loads, which is critical for repairing the network. CFNs should first be repaired (or replaced) before being brought into the network. The nodes that are simply disconnected from the network do not need to be specially repaired, and can work directly as long as they are reconnected to the network. Therefore, the reduction of CFNs is better both in the economic and the repair efficiency.

**Figure 5:**
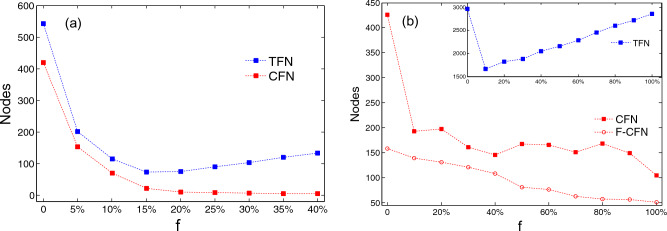
Failed nodes as a function of the fraction $f$ according to our strategy. (**a**) Simulation results of mail network; (**b**) Simulation results of Power-grid.

In order to better verify the effectiveness of the strategy, we conducted simulation calculations on two real networks: Email network^24^ and Power-grid^2^. The size of the Email network is $N = 1133$ and average connectivity degree of $\left\langle k \right\rangle = 9.62$. It can be found that the simulation results of the Email network are consistent with the simulation results of the synthetic network as shown in Fig. 5a. As the removal ratio $f$ increases, the number of TFNs first decreases and then increases, while the number of CFNs decreases monotonically. However, the result of the power-grid has new characteristics. The size of the Power-grid is $N = 4941$ and average connectivity degree of $\left\langle k \right\rangle = 2.67$, the process of cascading failure can be divided into 4 rounds, which is greater than the number of times of the email network and the scale-free network. The number of CNFs decreases with the increase of the removal ratio $f$, but it is not monotonously decreasing, the number of F-CNFs, which indicates the number of cascading failures in the first round, is monotonically decreasing as shown in Fig. 5b. This is because the total load of the network and the network size are positively correlated, proactively removing marginal nodes can reduce the impact of the first round of cascading failures, some nodes that should fail avoid cascading failure. However, the load on those nodes themselves is still on the verge of failure, therefore, the subsequent rounds of cascading may cause more serious failures.

As the number of proactively removed nodes increases, the total network load decreases, which indirectly increases the redundant capacity of each node and reduces the risk of this phenomenon. Therefore, the results of the cascading effects fluctuate as the proportion of proactive removals increases, but the overall trend remains downward. The simulation results of the power grid show that the effect of the proactive removal strategy of the large-scale network in the case of multiple rounds of cascading failures is reduced.

### Dynamic repair

Reasonable repair strategy is an important means to ensure network security. Many scholars have intensively studied the characteristics of network repair^25,26^, however, most studies ignore the effect of cascading effects in the repair process. When a failed node is repaired and rejoined to the damaged network, a secondary failure may occur because of overload^21^. Under the global load distribution model, it not only causes secondary failures of the repaired node itself but may also lead to a new round of cascading failures. Therefore, the correct repair order is a critical for network security.

According to the method mentioned above in this paper, the importance ranking based on the influence of nodes to the network energy fluctuation can be obtained, the destruction of higher-ranked nodes has a greater effect on the route of the initial network energy flow and results in greater energy confusion, as depicted in Fig. 1b. If the higher-ranked node is repaired, then the energy flow route in the damaged network can be restored to the original network state more quickly, but for the damaged network structure at this time, repairing the higher-ranked node causes severe energy confusion, if the redundant capacity of nodes cannot withstand the shock of chaotic energy, it may lead to secondary cascading failures or even a new cascading propagation. If a low-ranked node is preferentially repaired, the energy fluctuation caused by the repaired node is small, and the effect of restoring the initial network structure is also small. However, the redundant capacity of the node with small load is also small, and the impact resistance to energy shock is poor, and it is easy to cause secondary failures due to energy disturbances generated in the subsequent repair process.

**Figure 6:**
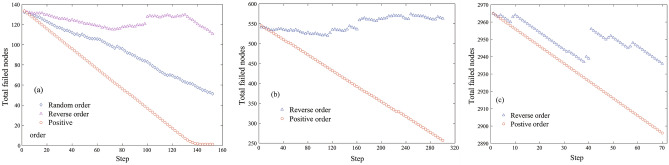
The variation of total number of failed nodes with step distance. (**a**) The synthetic network, (**b**) the real network of email, (**c**) the real network of power.

We assumed that at each time step, only one node is repaired and connected to the network. If the repaired node fails again or causes more nodes to fail due to cascade failure, all the newly failed nodes are individually sorted and added to the repair order. In Fig. 6a, based on the node ranking obtained by the proposed method, three repair strategies were simulated: (1) positive order, where nodes are repaired from the core to the periphery by node ranking; (2) reverse order, the repair order of nodes is opposite to the positive order; and (3) random order. The repair effect of the positive order is the best and no secondary failure occurs. The repair effect of the reverse effect is the worst, and a large number of repaired nodes results in secondary failures and even causes new cascading effects. If the network is attacked and a large number of nodes fail or are removed, according to Section II, the nodes leaving the network take away the energy of the network, and thus, the redundant capacity of the remaining nodes increases. When the repaired node is reconnected to the damaged network, a higher-level node (the core node) causes severe energy confusion to the damaged network. However, this energy confusion is essentially a correction that restores the energy flow path under the original network. It makes more load return to its original node; consequently, the increased redundant capacity can withstand the energy disturbance caused by the remaining redistributed loads. The earlier the highly ranked node is repaired, the more the risk of cascading failure is reduced. That is, the increased redundancy capacity can withstand energy disturbances caused by the remaining redistributed load. Therefore, positive order repair is the best. Figure 6b and c are the simulation results of the email network and power grid, respectively. Obviously, the simulation results of the real network and the synthetic network are consistent, positive order repair can well avoid secondary failure.

**Figure 7:**
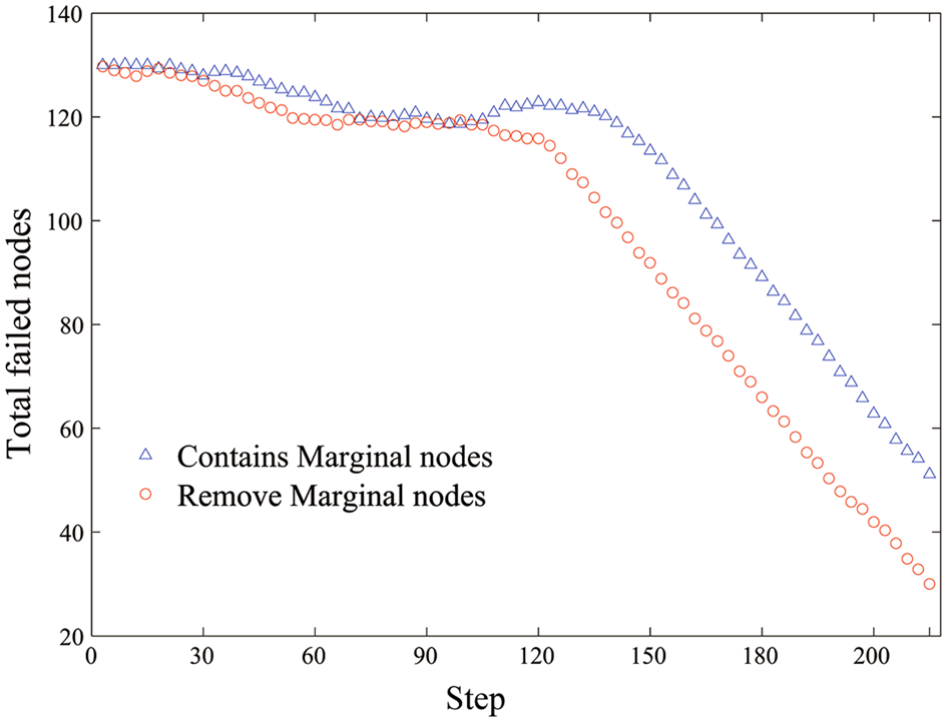
The variation of total number of failed nodes with step distance using reverse order repair strategy.

The impact of removing marginal nodes in the reverse repair strategy on the repair effect was further analyzed, as shown in Fig. 7. A large number of nodes repaired at the beginning had secondary failures, and even caused new cascading failures. Although the energy confusion caused by the repaired node is small, however, the energy flow in the damaged network deviated from the energy flow path of the original network. Therefore, a large number of repaired nodes again fails because of the inadequate redundant capacity, and effective repair was not achieved until the highly loaded nodes were repaired. After a certain inflection point, subsequent nodes can be repaired normally without secondary failures. This is because the inflection point and nodes after the point can properly restore the energy flow path of the original network, restore the consistency of capacity and load relationship of each node, and make subsequent repairs easier. In addition, the marginal nodes have no influence on the route of the energy flow in the network but increase the network load; therefore, the priority repair of marginal nodes increases the risk of cascading effects when higher-ranking nodes are repaired. The result is shown in Fig. 7 where the open circles curve is better than the open square curve.

In summary, under the global load distribution model, the principle of dynamic repair is as follows: (1) First, repair the nodes with the higher-ranked, most of which are also high-load nodes. This part of the nodes has a high redundancy capacity to resist energy shock, and the energy flow path of the original network can be restored more effectively; (2) It is a better strategy to repair all the inner ring nodes before repairing the marginal nodes. The repair of the marginal nodes under the damaged network structure has the risk of secondary failure, but it does not exist under the initial network structure, because the energy flow of the marginal node to the initial network only increases the load and does not change the energy flow path, it cannot correct the flow path of the damaged network load, which is not conducive to the recovery of the network.

## Conclusion

The initial information of the network is the basis for formulating attack strategies, defense strategies, and repair strategies. The existence of marginal nodes obscures the initial information of the network. Therefore, strategies based on the initial information of the network cannot achieve the expected results.

In this study, we first analyzed the relationship between nodes and network energy under the cascade model. The influence of the marginal nodes on the energy fluctuation of the network was analyzed, and two methods were proposed to screen the marginal nodes. Then, all nodes were sorted according to the intensity of the network energy confusion caused by removing the network nodes. The results were effectively applied in proactive defense and dynamic repair. Proactively removing marginal nodes can effectively reduce the effect of cascading failures without any negative effects. The effect of marginal nodes is the opposite when dynamically repairing the network, and repairing according to the positive order strategy can minimize the cascading risk during the repair process. However, the marginal nodes are only affected by the network structure. How to design a reasonable network structure is a question worth exploring. The proactive removal strategy only has an effect on the first round of cascading impact. How to find a suitable strategy to reduce the cascading impact during the entire failure process is a problem worth exploring. In summary, our study proved that based on the processed network information, more effective defense and repair strategies can be formulated to improve network security.

## Data Availability

The datasets used and/or analyzed during the current study available from the corresponding author on reasonable request.

## References

[1] Newman MEJ (2010). Networks: an introduction.

[2] Watts DJ, Strogatz SH (1998). Collective dynamics of ‘small-world’ networks. Nature.

[3] Barabasi A-L, Albert R (1999). Emergence of scaling in random networks. Science.

[4] Callaway DS (2000). Network robustness and fragility: Percolation on random graphs. Phys. Rev. Lett..

[5] Song CM, Havlin S, Makse HA (2005). Self-similarity of complex networks. Nature.

[6] Albert R, Barabasi AX (2000). Topology of evolving networks: local events and university. Nature.

[7] Dorogovtsev SN, Goltsev AV, Mendes JFF (2006). k-Core organization of complex networks. Phys. Rev. Lett..

[8] Wang JW (2013). Robustness of complex networks with the local protection strategy against cascading failures. Saf. Sci..

[9] Buldyrev SV, Parshani R, Paul G (2010). Catastrophic cascade of failures in interdependent networks. Nature.

[10] Yang R, Wang WX, Lai YC, Chen GR (2009). Optimal weighting scheme for suppressing cascades and traffic congestion in complex networks. Phys. Rev. E..

[11] Lai YC, Motter AE, Nishikawa T (2004). Attacks and cascades in complex networks. Lect. Notes Phys..

[12] Cai KQ, Zhang J, Du WB, Cao XB (2012). Analysis of the Chinese air route network as a complex network. Chin. Phy. B.

[13] Kumar R, Kumari S, Bala M (2021). Minimizing the effect of cascade failure in multilayer networks with optimal redistribution of link loads. J. Compl. Netw..

[14] Hu K, Hu T, Tang Y (2010). Model for cascading failures with adaptive defense in complex networks. Chin. Phys. B.

[15] Crucitti P, Latora V, Marchiori M (2004). Model for cascading failures in complex networks. Phys. Rev. E Stat. Nonlinear Soft Matter Phys..

[16] Shi R (2023). Power grid structure performance evaluation based on complex network cascade failure analysis. Energies.

[17] González SH, De La Mota IF (2021). Applying complex network theory to the analysis of Mexico city metro network (1969–2018). Case Stud. Trans. Policy.

[18] Qian B, Zhang N (2022). Topology and robustness of weighted air transport networks in multi-airport region. Sustainability.

[19] Wang JR, Wang JP, He Z, Xu HT (2015). Degree distribution and robustness of cooperative communication network with scale-free model. Chin. Phys..

[20] Motter AE (2004). Cascade control and defense in complex networks. Phys. Rev. Lett..

[21] Fu CQ, Wang Y, Gao Y (2017). Complex networks repair strategies: Dynamic models. Phys. A Stat. Mech. Appl..

[22] Fu CQ, Wang Y, Zhao K (2018). Complex networks under dynamic repair model. Phys. A Stat. Mech. Appl..

[23] Zhao L, Park K, Lai YC (2005). Tolerance of scale-free networks against attack-induced cascades. Phys. Rev. E.

[24] Xiaoping Su, Yurong S (2015). Levering neighborhood “structural holes” to identifying key spreaders in social networks. Acta Phys. Sin..

[25] Sun WM, Zeng A (2017). Target recovery in complex networks. Euro. Phys. J. B Condens. Matter Phys..

[26] Farr RS, Harer JL, Fink TMA (2014). Easily repairable networks: Reconnecting nodes after damage. Phys. Rev. Lett..

